# Repetitive DNA Restructuring Across Multiple *Nicotiana* Allopolyploidisation Events Shows a Lack of Strong Cytoplasmic Bias in Influencing Repeat Turnover

**DOI:** 10.3390/genes11020216

**Published:** 2020-02-19

**Authors:** Steven Dodsworth, Maïté S. Guignard, Oscar A. Pérez-Escobar, Monika Struebig, Mark W. Chase, Andrew R. Leitch

**Affiliations:** 1 School of Life Sciences, University of Bedfordshire, Luton LU1 3JU, UK; 2 School of Biological and Chemical Sciences, Queen Mary University of London, London E1 4NS, UK; M.Guignard@kew.org (M.S.G.); M.S.Struebig@kent.ac.uk (M.S.); 3 Royal Botanic Gardens, Kew, Richmond TW9 3AB, UK; O.PerezEscobar@kew.org (O.A.P.-E.); m.chase@kew.org (M.W.C.); 4 Department of Environment and Agriculture, Curtin University, Bentley 6102, Western Australia, Australia

**Keywords:** polyploidy, allopolyploidisation, repeats, retroelements, genome reorganisation, diploidisation, nuclear-cytoplasmic interaction hypothesis, genome evolution

## Abstract

Allopolyploidy is acknowledged as an important force in plant evolution. Frequent allopolyploidy in *Nicotiana* across different timescales permits the evaluation of genome restructuring and repeat dynamics through time. Here we use a clustering approach on high-throughput sequence reads to identify the main classes of repetitive elements following three allotetraploid events, and how these are inherited from the closest extant relatives of the maternal and paternal subgenome donors. In all three cases, there was a lack of clear maternal, cytoplasmic bias in repeat evolution, i.e., lack of a predicted bias towards maternal subgenome-derived repeats, with roughly equal contributions from both parental subgenomes. Different overall repeat dynamics were found across timescales of <0.5 (*N. rustica* L.), 4 (*N. repanda* Willd.) and 6 (*N. benthamiana* Domin) Ma, with nearly additive, genome upsizing, and genome downsizing, respectively. Lower copy repeats were inherited in similar abundance to the parental subgenomes, whereas higher copy repeats contributed the most to genome size change in *N. repanda* and *N. benthamiana*. Genome downsizing post-polyploidisation may be a general long-term trend across angiosperms, but at more recent timescales there is species-specific variance as found in *Nicotiana*.

## 1. Introduction

Allopolyploidy is pervasive in flowering plants, having occurred multiple times throughout angiosperm evolutionary history and recurrently in most clades [1]. Allopolyploidy occurs where genome duplication and hybridisation happen in concert. This has led to an appreciation of polyploidy (or whole genome duplication) as an important driving force in plant evolution, with the potential for immediate disadvantages of neopolyploidy ultimately leading to evolutionary novelty and ecological persistence [2,3,4,5,6]. The combination of two divergent subgenomes within one nucleus, and the redundancy of DNA sequences, leads to complicated post-polyploidisation restructuring of the polyploid genome as it is diploidised [7]. In *Nicotiana* (Solanaceae), recurrent polyploidisation has led to allotetraploid species of different ages, making this an excellent model system for investigating allopolyploidisation through time [8,9,10,11,12,13]. This allows us to test whether similar outcomes occur through allopolyploidisation within one genus, over different time scales, or whether post-polyploidisation changes are instead species-specific. 

**Figure 1:**
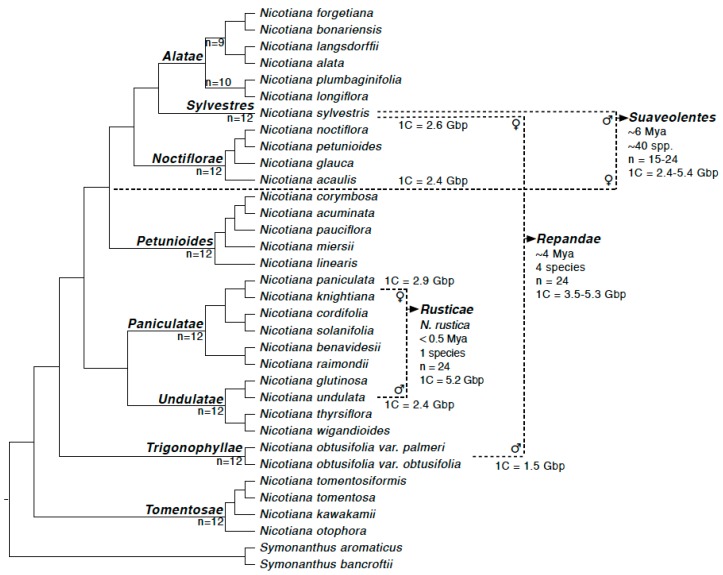
The evolutionary history of the three allotetraploid sections studied here (*Rusticae*, *Repandae* and *Suaveolentes*) including chromosome numbers, genome sizes and direction of hybridisation. Figure adapted from [19,21,22].

A combination of genomic, cytogenetic and phylogenetic work over the past two decades has established the parentage and age of the allotetraploid species of *Nicotiana* [14,15,16,17,18]. At the younger end of the spectrum (<0.5 Ma) is the commercial species, *N. tabacum* L., the common smoking tobacco. The closest extant relatives of the parental lineages for *N. tabacum* (section *Nicotiana*) are well-established as being *N. tomentosiformis* Goodsp. (paternal; T-genome donor) and *N. sylvestris* Speg. (maternal; S-genome donor) [9,14] (Figure 1). Young polyploids are also found within the sections *Rusticae* and *Polydicliae*. *Nicotiana rustica* is formed from *N. undulata* Ruiz & Pav. (paternal) and *N. paniculata* L. (maternal) in <0.5 Ma ([14,19]; Figure 1). Section *Repandae* formed approximately 4 Ma, between *N. obtusifolia* M.Martens & Galeotti (paternal) and *N. sylvestris* (maternal) and consists of four taxa (Figure 1): *N. repanda*, *N. stocktonii* Brandegee, *N. nesophila* I.M.Johnst. and *N. nudicaulis* S.Watson [11,14,18,19]. Finally, the largest section of polyploid taxa is section *Suaveolentes* (approximately 50 species), which is also the oldest section of allotetraploids, appearing at approximately 6–7 Ma [18,20]. The parentage of this section is probably more complex and likely involves *N. sylvestris* (paternal sub-genome donor) and a homoploid hybrid as the maternal subgenome donor (between sections *Noctiflorae* and *Petunioides*; Figure 1). The section *Suaveolentes* also includes the important model *N. benthamiana* that is used extensively for plant-viral interaction studies. Recent genomic analysis using gene-based phylogenetic trees indicated that approximately 1.4 Gbp of the *N. benthamiana* genome is attributable to *N. noctiflora* Hook. as the maternal parent [20] and thus, we consider *N. noctiflora* as a close relative of the maternal parent for *N. benthamiana*. 

Genome restructuring occurs post-polyploidisation, perhaps as one result of “genomic shock”, the combination of two divergent subgenomes within one nucleus (McClintock, 1984), although this phenomenon is more pronounced in herbaceous annuals than in perennials and woody groups. The nuclear-cytoplasmic interaction hypothesis (NCI) proposes to explain some of the patterns seen in the formation of allopolyploids. The paternal genome is considered as alien DNA within the context of the maternal cytoplasm, which could therefore lead to vulnerability of the paternal sub-genome, and ultimately to its paternal-specific degradation [23]. Previous results have shown a bias in the loss of paternal DNA (T-genome) for *Nicotiana tabacum* [9,10,23]. Similarly, the recent draft genome of *N. rustica* has suggested that approximately 59% originated from the maternal genome donor (*N. knightiana*/*N. paniculata*) versus 41% from the paternal genome donor, using a k-mer analysis of sequence data [24]. 

Here we examine the parental contribution to the allotetraploid genome across three allotetraploidisation events of different ages: (i) *N. rustica* (<0.5 Ma; section *Rusticae*); (ii) *N. repanda* (~4 Ma; section *Repandae*); and (iii) *N. benthamiana* (~6 Ma; section *Suaveolentes*). We specifically ask the following questions:

1. How do repeat dynamics vary across polyploidisation events of different ages?
2. Is there a higher contribution of maternal subgenome DNA to the allopolyploid genome, reflected in repetitive element abundances?
3. If present, do these biases vary with the age of the allopolyploid, and/or phylogenetic distance of progenitor subgenomes?

## 2. Materials and Methods

### 2.1. Illumina Sequencing Data

Genomic libraries were prepared and sequenced for *N. noctiflora* [SRR11051011], *N. undulata* [SRR11051010], *N. paniculata* [SRR11051009] and *N. rustica* [SRR11051008] as described in [25], using Illumina TruSeq PCR-free kits and 150 bp PE Illumina sequencing. Read data for the remaining taxa were downloaded from the NCBI Sequence Read Archive as follows: *Nicotiana sylvestris* [SRR343066]; *Nicotiana obtusifolia* [SRR452993]; *Nicotiana repanda* [SRR453021]; *Nicotiana benthamiana* [SRR7540368]. Reads were quality filtered (Phred score minimum >10, maximum 3 Ns) and trimmed to the same length (91 bp). Higher quality filtering can lead to biased estimates of repeat abundance, due to typically lower quality scores for AT/GC rich sequences such as satellite repeats. 

### 2.2. Clustering of Read Data

In each case, read subsets representing a genome proportion of 2% (0.02 × coverage) were used to estimate the repetitive element abundance in each diploid and allotetraploid genome. These were combined to create three final datasets as follows: (i) *Rusticae* (*N. rustica* + ♂*N. undulata* + ♀*N. paniculata*); (ii) *Repandae* (*N. repanda* + ♂*N. obtusifolia* +♀*N. sylvestris*); and (iii) *Suaveolentes* (*N. benthamiana* + ♂*N. sylvestris* + ♀*N. noctiflora*). Reads were prefixed with five-letter sample-specific codes, and comparative clustering was run on each dataset using RepeatExplorer2, using default settings [26,27].

### 2.3. Statistical Analyses

Statistical analyses and graphical plots were performed in R version 3.3.0 [28]. Contaminating clusters were removed (sequence artefacts, organellar DNA), as well as clusters with fewer than 10 reads (per species) and species-specific clusters. The expected cluster size for each tetraploid species was calculated as the sum of the abundance in each parent. The cumulative deviation from expectation was calculated and plotted against the cumulative expected cluster size in order to visualise repeat dynamics across the range of cluster sizes, as in [11]. Linear regression was fitted to test parental contributions to tetraploid cluster size. Cluster sizes (number of reads) were natural log-transformed. Three-dimensional plots were plotted using the *plot3D* package [29] and the *car* package [30] was used to compare the parental regression slopes. In all analyses, residuals were viewed to ensure that model assumptions were met. 

## 3. Results

### 3.1. Repeat Dynamics in Polyploids of 0.5–6 Ma

**Figure 2:**
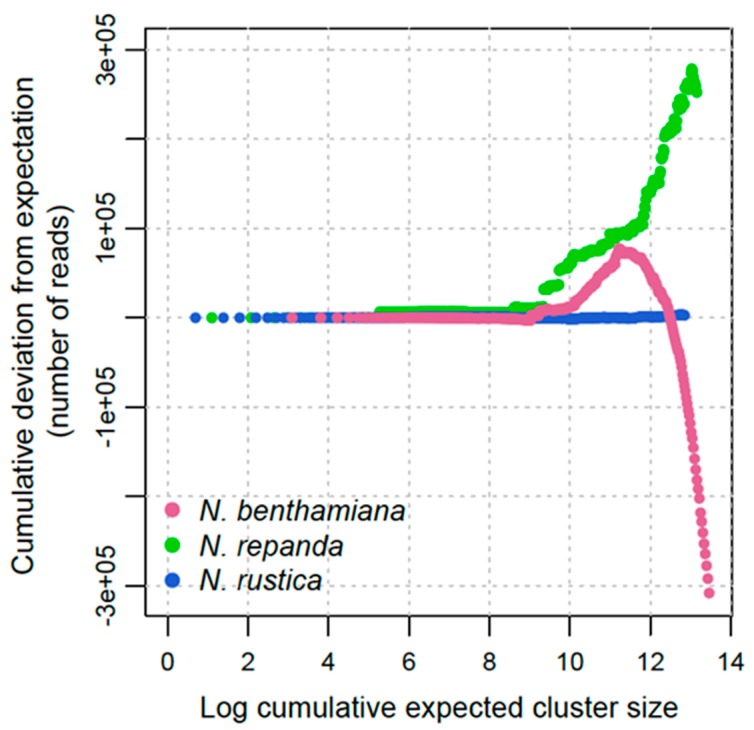
Repeat dynamics for *N. rustica* (section *Rusticae*), *N. repanda* (section *Repandae*) and *N. benthamiana* (section *Suaveolentes*). Curves for each accession represent the absolute cumulative deviation from expectation (sum of parental values). Clusters are ranked from smallest (left) to largest (right), plotted on a natural log scale.

To broadly compare the repeat dynamics across polyploidisation events, we compared the deviation from expected abundance for a range of cluster sizes from ten reads upwards (Figure 2). These represent different classes of repetitive elements. The expected cluster size is the sum of the cluster size in each of the two parental subgenomes for each allotetraploid. Deviation above zero represents an increase in repeat abundance from expectation and below zero the opposite. 

In the young tetraploid *Nicotiana rustica* there is little deviation from expectation across the range of repeat sizes (Figure 2). This indicates that *N. rustica* has faithfully maintained repetitive DNA without significant sequence divergence or genome size change. Comparing the deviation from expectation at the same scale across polyploidisation events (Figure 2), shows that any deviation is minimal compared with the alterations that occur in older tetraploids. 

Over longer timescales, deviation from simple expectation is much greater in both of the older tetraploid groups for sections *Repandae* (~4 Ma) and *Suaveolentes* (~6 Ma). In both older tetraploids, there is limited deviation from expected cluster size at the small-medium sizes (up to about 5000 reads). As cluster size increases, however, these higher abundance elements have significantly differed from expectation in both cases. For *N. repanda* the larger abundance elements are more represented than expected, resulting in a final genome size that is larger than expected (Figure 2). For *N. benthamiana*, there is an initial increase above expectation for some larger clusters (mid-size repeats at Log 10-11), but this quickly becomes a decrease, showing appreciable genome downsizing from expectation. This appears to be almost entirely due to deletion of higher abundance repeat elements (Figure 2). 

### 3.2. Parental Contribution to Allotetraploid Genomes

**Figure 3:**
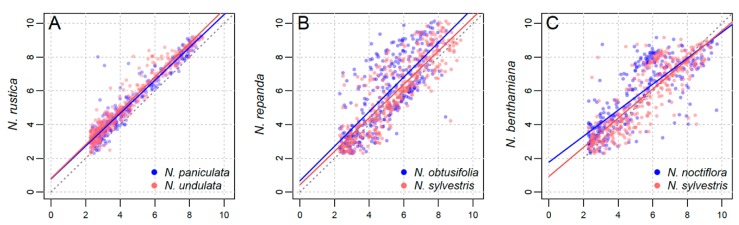
Regression analyses of cluster size (read number) in the parental subgenomes versus the tetraploid genome, natural log-transformed. (**A**) *N. rustica* against *N. paniculata* (maternal; blue) and *N. undulata* (paternal; red). (**B**) *N. repanda* against *N. obtusifolia* (paternal; blue) and *N. sylvestris* (maternal; red). (**C**) *N. benthamiana* against *N. noctiflora* (maternal; blue) and *N. sylvestris* (paternal; red).

**Figure 4:**
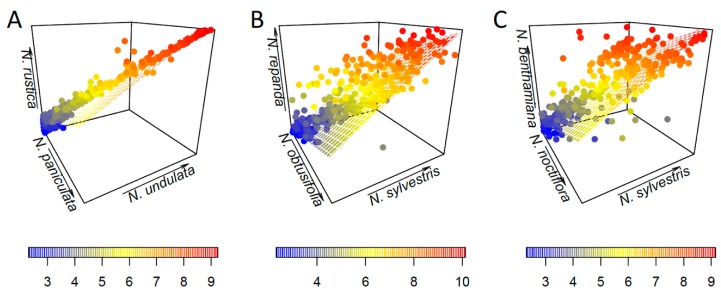
Results of 3D regression analyses. Cluster size is coloured from blue (low) to red (high). (**A**) *N. rustica* against *N. paniculata* (maternal) and *N. undulata* (paternal). (**B**) *N. repanda* against *N. obtusifolia* (paternal) and *N. sylvestris* (maternal). (**C**) *N. benthamiana* against *N. noctiflora* (maternal) and *N. sylvestris* (paternal).

For each tetraploid, we conducted a series of regression analyses to explore the relative contribution of the paternal vs. maternal parents (Figure 3 and Figure 4). In all cases, there is no clear difference between the maternal and paternal contribution (Figure 3), with no obvious pattern across the range of cluster sizes (Figure 4). 

In the youngest polyploid, *N. rustica*, there is a stronger correlation between the parental cluster size and that of the tetraploid (Figure 3A), with a statistically significant difference between the slope of the two parental regression lines (*p* = 0.0201). For both older tetraploids, *N. repanda* (*p* = 0.0002) and *N. benthamiana* (*p* = 0.0046) the slopes were found to be significantly different between the maternal and paternal progenitors (Table 1; Table S1), although visually there is a lack of obvious pattern or direction in the regressions (Figure 3B,C). Untransformed plots (Figure S1) and LOESS plots (Figure S2) are additionally shown in Supplementary Materials. 

Plotting the regression results in 3D as an alternative visualisation also showed no clear pattern between maternal and paternal parents (Figure 4; Table S2). In the case of *N. rustica*, the cluster sizes have a much higher correlation between parents and polyploid, and hence fit closer to the regression plane (Figure 4A). For older polyploids, *N. repanda* (Figure 4B) and *N. benthamiana* (Figure 4C), there is dispersion across the range of cluster sizes and around the regression plane. Summary statistics for each dataset are given in Table S3.

## 4. Discussion

### 4.1. Repeat Restructuring across 0.5–6 Ma Timescales

In the youngest allotetraploid, *N. rustica* (Figure 2), repeat abundances are close to the sum of abundances expected from both parental donors. *Nicotiana rustica* has a genome size of 1C = 5.2 Gbp [22], which is close to the sum (5.3 Gbp) of the extant parental lineages *N. paniculata* (2.9 Gbp) and *N. undulata* (2.4 Gbp). It also has retained all 24 pairs of chromosomes (12 pairs from each subgenome). Previous studies using GISH (genomic *in-situ* hybridisation) showed clear additivity for both parental subgenomes and a lack of any clear genomic translocations [8,31]. Additionally, the distribution of several tandem repeats of the HRS60 family showed chromosomal locations concordant with their distribution in the parental genomes, apart from one from the *N. undulata* subgenome [31]. For ribosomal DNA, sequence conversion of the 18-5.8-26S locus is towards the *N. undulata* type (approx. 80% of sequences), the paternal genome donor [32], the same pattern found for *N. tabacum*. Recent studies in *N. tabacum* found there is much greater restructuring, including genomic translocations and significant loss of repetitive DNA from the paternal T-genome. Similar results were also found in synthetic lines of *N. tabacum* in the fourth generation [9,10,23]. 

In older tetraploids, repeat dynamics are much more variable (Figure 2), which likely reflects greater restructuring of the genome and turnover of repetitive elements. Species of section *Repandae* have retained all chromosomes (*n* = 24), ca. 4 Ma [18], but translocations identified via GISH [11] show genome exchange and reorganisation. In *N. repanda*, there is significant genome upsizing from the expected sum of parental subgenomes, of around 29% (observed 1C = 5.3 Gbp versus expected 1C = 4.1 Gbp) [11,13,22]. This genome size change is mostly due to an increase in high-abundance repeats, including Ty3/Gypsy chromovirus retroelements [13], and the same trajectory of repeat accumulation is found in *N. stocktonii* and *N. nesophila* [11]. Repeat dynamics are, however, to an extent species-specific, because *N. nudicaulis* has experienced overall genome downsizing (−14%), and its repeat abundances/types are much closer to expectation [11,13].

Section *Suaveolentes* is the oldest (ca. 6 Ma; [18]) and most species-rich [33] group of tetraploids in *Nicotiana*. In this section, most species have genome sizes that represent stark genome downsizing [34], and *N. benthamiana* has a genome size of 1C = 3.3 Gbp. This is in addition to a reduction in chromosome number (from *n* = 24 to *n* = 15 in some species), likely the result of descending dysploidy [34], not necessarily correlated with genome-size reduction. Ancestrally, section *Suaveolentes* has *n* = 24, but *N. benthamiana* has *n* = 18, 19. Such extensive genome reorganisation as part of the diploidisation process could potentially mask events that occurred shortly after allotetraploidisation at approx. 6 Ma [18]. It would be important to sample repeat types/abundances in several of the species with the ancestral (or nearly) chromosome numbers (there are six species with *n* = 23, 24) but among these, there is a wide range of genome sizes, 1C = 5.45–2.87 Gbp. Given the number of taxa in section *Suaveolentes* (ca. 40–50 species), investigating how repeat dynamics vary in this group will be central to discovering how it relates (or not) to chromosomal changes and genome sizes in a descending dysploid series (*n* = 15–24). In other groups, particularly Brassicaceae, there is extensive dysploidy post-polyploidisation [35], and this process is likely responsible for much of the diversification of angiosperms [7].

### 4.2. Lack of a Clear Parental Bias in Repeat Loss/Retention 

As part of the “genomic shock” experienced by combining two divergent subgenomes within one neoallopolyploid nucleus, there are predictions regarding the level of genome reorganisation, and sequence loss/retention based on the direction of the cross. The maternal subgenome is expected to be favoured, relative to the paternal subgenome, due to their compatibility within the maternal cytoplasm. This potentially leads to specific degradation of various elements from the paternal subgenome, as predicted by the NCI hypothesis. In *N. tabacum* this was found to be the case, including synthetic lines, whereby the T-genome from *N. tomentosiformis* is significantly reduced relative to the S-genome from *N. sylvestris* [9,10]. However, not all lines showed genomic translocations, as predicted by NCI, which are therefore assumed to be more stochastic and not essential to polyploid success [23]. 

In the three allotetraploids studied here, we found no significant evidence of maternal bias in the repeat abundances (Figure 3 and Figure 4). *Nicotiana rustica* has a much closer correlation between parental cluster size and cluster size (Figure 3A and Figure 4A), but its two parents are much more closely related than those of *N. tabacum* (Figure 1). This could give post-tetraploid diploidisation more stochasticity due to greater overall similarity of the two parental genomes (i.e., they are simply too similar for there to be a noticeable effect favouring one subgenome over the other). Additionally, the slopes of the regression lines were not found to be statistically different (Table 1). This reveals a faithful inheritance of most repeats from both parents, *N. undulata* and *N. paniculata*, and a lack of maternal cytoplasmic bias. Previously, this was suggested on the basis of a lack of genomic translocations, particularly involving perturbation of the paternal subgenome [23,31]. It is possible that if overcoming a greater level of genomic shock involves transgenomic translocations, then the effects of genomic incompatibility are greater, leading to a maternal bias, whereas if the two parental genomes are more similar then incompatibility is a much more subtle factor. We would argue that the ages of the two older allotetraploids also makes detecting parental biases difficult to impossible–too much genomic change has taken place post-polyploidisation to detect parental biases that might have been operating at earlier stages in the process. 

For *N. repanda* and *N. benthamiana*, correlation between cluster size in parental lineages and tetraploids is much less clear (Figure 3 and Figure 4), which reflects the longer timescales over which these tetraploid genomes have diverged. Slopes of parental regressions were significantly different in both cases (Figure 3B,C; Table 1), but there is no clear directionality, e.g., favourable retention of maternal repeat abundances. In both cases, repeat changes in the allotetraploid are mostly a result of changes in higher copy sequences, but these are present in both parental subgenomes and subject to overall similar pressures. In the case of *N. repanda* this is overall genome upsizing, whereas in the case of *N. benthamiana* there is significant genome downsizing (Figure 2). 

## 5. Conclusions 

Overall our analyses show extensive repeat restructuring over longer time frames in sections *Repandae* and *Suaveolentes*, with the former experiencing genome upsizing and the latter significant genome downsizing. In the younger polyploid *N. rustica* we show little deviation from the expected sum of parental subgenomes. We did not find significant evidence of overall maternal bias in repeat retention as found previously for *N. tabacum* across the three separate allotetraploidisation events of <0.5 (*N. rustica*) 4 (*N. repanda*) and 6 (*N. benthamiana*) Ma respectively. Future work comparing the signatures of sequence retention in repetitive DNA, dark matter (i.e., degraded repeats) and gene space will be important to understand whether different processes govern the evolution of different sequence types post-polyploidisation and how these contribute to genome reorganisation. 

## Figures and Tables

**Table 1:** Results of *car* package testing whether the parental slopes are equal for each polyploid taxon.

Polyploid	Parents	Sum of Sq.	*F* Stat	*p*-Value
*N. benthamiana*	*N. noctiflora*, *N. sylvestris*	8.617	8.138	0.0046
*N. repanda*	*N. obtusifolia*, *N. sylvestris*	14.297	13.840	0.0002
*N. rustica*	*N. paniculata*, *N. undulata*	0.491	5.451	0.0201

## Supplementary Materials

The following are available online at https://www.mdpi.com/2073-4425/11/2/216/s1, Table S1: Linear regression output. Repetitive element abundance (read number) was natural-log transformed; Table S2: Linear regression output in relation to 3D plots in Figure 4; Table S3: Summary statistics of the three polyploid datasets for reads ≥10; Figure S1: Regression analyses of cluster size (read number) in the parental subgenomes versus the tetraploid genome, untransformed. 2:1 and 1:1 lines shown; Figure S2: Regression analyses with LOESS lines fitted.

## References

[1] Wendel J.F. (2015). The wondrous cycles of polyploidy in plants. Am. J. Bot..

[2] Wood T.E., Takebayashi N., Barker M.S., Mayrose I., Greenspoon P.B., Rieseberg L.H. (2009). The frequency of polyploid speciation in vascular plants. Proc. Natl. Acad. Sci. USA.

[3] Mayrose I., Zhan S.H., Rothfels C.J., Magnuson-Ford K., Barker M.S., Rieseberg L.H., Otto S.P. (2011). Recently formed polyploid plants diversify at lower rates. Science.

[4] Landis J.B., Soltis D.E., Li Z., Marx H.E., Barker M.S., Tank D.C., Soltis P.S. (2018). Impact of whole-genome duplication events on diversification rates in angiosperms. Am. J. Bot..

[5] Eric Schranz M., Mohammadin S., Edger P.P. (2012). Ancient whole genome duplications, novelty and diversification: The WGD Radiation Lag-Time Model. Curr. Opin. Plant Biol..

[6] Tank D.C., Eastman J.M., Pennell M.W., Soltis P.S., Soltis D.E., Hinchliff C.E., Brown J.W., Sessa E.B., Harmon L.J. (2015). Nested radiations and the pulse of angiosperm diversification: Increased diversification rates often follow whole genome duplications. New Phytol..

[7] Dodsworth S., Chase M.W., Leitch A.R. (2016). Is post-polyploidization diploidization the key to the evolutionary success of angiosperms?. Bot. J. Linn. Soc..

[8] Lim K.Y., Matyasek R., Kovarik A., Leitch A.R. (2004). Genome evolution in allotetraploid *Nicotiana*. Biol. J. Linn. Soc..

[9] Renny-Byfield S., Chester M., Kovarik A., Le Comber S.C., Grandbastien M.-A., Deloger M., Nichols R.A., Macas J., Novak P., Chase M.W. (2011). Next generation sequencing reveals genome downsizing in allotetraploid *Nicotiana tabacum*, predominantly through the elimination of paternally derived repetitive DNAs. Mol. Biol. Evol..

[10] Renny-Byfield S., Kovařík A., Chester M., Nichols R.A., Macas J., Novák P., Leitch A.R. (2012). Independent, rapid and targeted loss of highly repetitive DNA in natural and synthetic allopolyploids of *Nicotiana tabacum*. PLoS ONE.

[11] Dodsworth S., Jang T.S., Struebig M., Chase M.W., Weiss-Schneeweiss H., Leitch A.R. (2017). Genome-wide repeat dynamics reflect phylogenetic distance in closely related allotetraploid *Nicotiana* (Solanaceae). Plant Syst. Evol..

[12] Clarkson J.J., Lim K.Y., Kovarik A., Chase M.W., Knapp S., Leitch A.R. (2005). Long-term genome diploidization in allopolyploid *Nicotiana* section *Repandae* (Solanaceae). New Phytol..

[13] Renny-Byfield S., Kovarik A., Kelly L.J., Macas J., Novak P., Chase M.W., Nichols R.A., Pancholi M.R., Grandbastien M.-A., Leitch A.R. (2013). Diploidization and genome size change in allopolyploids is associated with differential dynamics of low- and high-copy sequences. Plant J..

[14] Clarkson J.J., Knapp S., Garcia V.F., Olmstead R.G., Leitch A.R., Chase M.W. (2004). Phylogenetic relationships in *Nicotiana* (Solanaceae) inferred from multiple plastid DNA regions. Mol. Phylogenet. Evol..

[15] Clarkson J.J., Kelly L.J., Leitch A.R., Knapp S., Chase M.W. (2010). Nuclear glutamine synthetase evolution in *Nicotiana*: Phylogenetics and the origins of allotetraploid and homoploid (diploid) hybrids. Mol. Phylogenet. Evol..

[16] Kelly L.J., Leitch A.R., Clarkson J.J., Hunter R.B., Knapp S., Chase M.W. (2010). Intragenic recombination events and evidence for hybrid speciation in *Nicotiana* (Solanaceae). Mol. Biol. Evol..

[17] Kelly L.J., Leitch A.R., Clarkson J.J., Knapp S., Chase M.W. (2013). Reconstructing the complex evolutionary origin of wild allopolyploid tobaccos (*Nicotiana* section *Suaveolentes*). Evolution.

[18] Clarkson J.J., Dodsworth S., Chase M.W. (2017). Time-calibrated phylogenetic trees establish a lag between polyploidisation and diversification in *Nicotiana* (Solanaceae). Plant Syst. Evol..

[19] Knapp S., Chase M.W., Clarkson J.J. (2004). Nomenclatural changes and a new sectional classification in *Nicotiana* (Solanaceae). Taxon.

[20] Schiavinato M., Marcet-houben M., Dohm J.C., Programme G., Barcelona T. (2019). Parental origin of the allotetraploid tobacco *Nicotiana benthamiana*. Plant J..

[21] Dodsworth S., Kovarik A., Grandbastien M.-A., Leitch I.J., Leitch A.R., Ivanov N.V. (2020). Repetitive DNA dynamics and polyploidization in the genus *Nicotiana* (Solanaceae). The Tobacco Genome.

[22] Leitch I.J., Hanson L., Lim K.Y., Kovarik A., Chase M.W., Clarkson J.J., Leitch A.R. (2008). The ups and downs of genome size evolution in polyploid species of *Nicotiana* (Solanaceae). Ann. Bot..

[23] Leitch A.R., Lim K.Y., Skalicka K., Kovarik A., Cigma A.A., Durante M. (2006). Nuclear cytoplasmic interaction hypothesis and the role of translocations in *Nicotiana* allopolyploids. Radiation Risk Estimates in Normal and Emergency Situations.

[24] Sierro N., Battey J.N.D., Bovet L., Liedschulte V., Ouadi S., Thomas J., Broye H., Laparra H., Vuarnoz A., Lang G. (2018). The impact of genome evolution on the allotetraploid *Nicotiana rustica*—An intriguing story of enhanced alkaloid production. BMC Genom..

[25] Dodsworth S., Guignard M.S., Christenhusz M.J.M., Cowan R.S., Knapp S., Maurin O., Struebig M., Leitch A.R., Chase M.W., Forest F. (2018). Potential of herbariomics for studying repetitive DNA in angiosperms. Front. Ecol. Evol..

[26] Novák P., Neumann P., Macas J. (2010). Graph-based clustering and characterization of repetitive sequences in next-generation sequencing data. BMC Bioinform..

[27] Novák P., Neumann P., Pech J., Steinhaisl J., Macas J. (2013). RepeatExplorer: A Galaxy-based web server for genome-wide characterization of eukaryotic repetitive elements from next-generation sequence reads. Bioinformatics.

[28] Development Core Team (2016). A Language and Environment for Statistical Computing.

[29] Soetaert K. (2014). Plotting Multi-Dimensional Data.

[30] Fox J., Weisberg S., Adler D., Bates D.M., Baud-Bovy G., Ellison S., Firth D., Friendly M., Gorjanc G., Graves S. (2014). An R Companion to Applied Regression.

[31] Lim K.Y., Matyasek R., Kovarik A., Fulnecek J., Leitch A.R. (2005). Molecular cytogenetics and tandem repeat sequence evolution in the allopolyploid *Nicotiana rustica* compared with diploid progenitors *N. paniculata* and *N. undulata*. Cytogenet. Genome Res..

[32] Matyasek R., Lim K.Y., Kovarik A., Leitch A.R. (2003). Ribosomal DNA evolution and gene conversion in *Nicotiana rustica*. Heredity.

[33] Chase M.W., Christenhusz M.J.M., Conran J.G., Dodsworth S., Medeiros de Assis F.N., Felix L.P., Fay M.F. (2018). Unexpected diversity of Australian tobacco species (*Nicotiana* section *Suaveolentes*, Solanaceae). Curtis Bot. Mag..

[34] Dodsworth S. (2015). Genome Skimming for Phylogenomics. Ph.D. Thesis.

[35] Mandáková T., Lysak M.A. (2018). Post-polyploid diploidization and diversification through dysploid changes. Curr. Opin. Plant Biol..

