# Supplementary material for: Repetitive DNA Restructuring Across Multiple Nicotiana Allopolyploidisation Events Shows a Lack of Strong Cytoplasmic Bias in Influencing Repeat Turnover
Source: Genes (Basel). 2020 Feb 19;11(2):216. doi: 10.3390/genes11020216 (PMC7074350; doi:10.3390/genes11020216)

Table S1. Linear regression output, regressing the polyploid offspring on each parent taxon. Data were natural-log transformed. See also Fig. 3.

A) *N. rustica*: residual SE= 0.5442 on 336, adjusted R2 = 0.9216

	Estimate	CI 2.5%	CI 97.5%	Std. Error	t value	Pr(>|t|)
Intercept	0.771	0.62	0.92	0.07684	10.03	< 0.0001
N. paniculata	0.975	0.94	1.01	0.01548	62.97	< 0.0001

N. rustica: residual SE= 0.49 on 336 df, adjusted R2 = 0.9365

	Estimate	CI 2.5%	CI 97.5%	Std. Error	t value	Pr(>|t|)
Intercept	0.804	0.67	0.94	0.07	11.77	< 0.0001
N. undulata	1.002	0.97	1.03	0.01	70.49	< 0.0001

B) *N. repanda*: residual SE= 1.385 on 334 df, adjusted R2= 0.5869

	Estimate	CI 2.5%	CI 97.5%	Std. Error	t value	Pr(>|t|)
Intercept	0.667	0.18	1.16	0.25	2.67	0.0079
N. obtusifolia	1.025	0.93	1.12	0.05	21.84	< 0.0001

N. repanda: residual SE= 1.113 on 334 df, adjusted R2= 0.7331

	Estimate	CI 2.5%	CI 97.5%	Std. Error	t value	Pr(>|t|)
Intercept	0.430	0.06	0.80	0.19	2.28	0.0233
N. sylvestris	0.991	0.93	1.06	0.03	30.35	< 0.0001

C) *N. benthamiana*: residual SE= 1.239 on 307 df, adjusted R2=0.6178

	Estimate	CI 2.5%	CI 97.5%	Std. Error	t value	Pr(>|t|)
Intercept	1.770	1.39	2.15	0.19	9.27	< 0.0001
N. noctiflora	0.770	0.70	0.84	0.03	22.33	< 0.0001

N. benthamiana: residual SE= 1.105 on 307 df; adjusted R2= 0.6963

	Estimate	CI 2.5%	CI 97.5%	Std. Error	t value	Pr(>|t|)
Intercept	0.924	0.55	1.30	0.19	4.83	< 0.0001
N. sylvestris	0.864	0.80	0.93	0.03	26.59	< 0.0001

Table S2. Linear regression output, regressing the polyploid taxon on both parents. Data were natural-log transformed. See also 3D plots in Figure 4.

A) *N. rustica*: residual SE= 0.3001 on 335 df, adjusted R2 = 0.9762

	Estimate	CI 2.5%	CI 97.5%	Std. Error	t value	Pr(>|t|)
Intercept	0.566	0.48	0.65	0.043	13.160	< 0.0001
N. paniculata	0.473	0.43	0.51	0.020	23.680	< 0.0001
N. undulata	0.565	0.53	0.61	0.020	27.750	< 0.0001

B) *N. repanda*: residual SE= 1.016 on 333 df, adjusted R2= 0.7775

	Estimate	CI 2.5%	CI 97.5%	Std. Error	t value	Pr(>|t|)
Intercept	-0.239	-0.61	0.14	0.19	-1.253	< 0.0001
N. obtusifolia	0.411	0.31	0.51	0.050	8.221	< 0.0001
N. sylvestris	0.733	0.65	0.82	0.043	16.941	< 0.0001

C) *N. benthamiana*: residual SE=1.029 on 306 df, adjusted R2=0.7365

	Estimate	CI 2.5%	CI 97.5%	Std. Error	t value	Pr(>|t|)
Intercept	0.772	0.42	1.13	0.18	4.294	< 0.0001
N. noctiflora	0.327	0.23	0.42	0.047	6.917	< 0.0001
N. sylvestris	0.589	0.49	0.69	0.050	11.805	< 0.0001

Table S3. Summary statistics of the three datasets, with reads ≥10.

N. rustica		*N. paniculata*		*N. undulata*	
Min.	10	Min.	10	Min.	10
1st Qu.	39	1st Qu.	19	1st Qu.	17
Median	102	Median	49.5	Median	46.5
Mean	1094	Mean	578	Mean	502.1
3rd Qu.	1126	3rd Qu.	442.5	3rd Qu.	357
Max.	10377	Max.	5894	Max.	6413

N. sylvestris		*N. repanda*		*N. obtusifolia*	
Min.	10	Min.	10	Min.	10
1st Qu.	56.75	1st Qu.	48	1st Qu.	39
Median	226.5	Median	398.5	Median	170.5
Mean	998.5	Mean	2085	Mean	479.9
3rd Qu.	1017	3rd Qu.	1903	3rd Qu.	570.5
Max.	10827	Max.	25078	Max.	4657

N. sylvestris		*N. noctiflora*		*N. Benthamiana*	
Min.	10	Min.	10	Min.	10
1st Qu.	50	1st Qu.	24	1st Qu.	47
Median	311	Median	155	Median	363
Mean	1133	Mean	1140	Mean	1279
3rd Qu.	1204	3rd Qu.	716	3rd Qu.	2063
Max.	11094	Max.	16597	Max.	9491

Figure S1. Regression analyses of cluster size (read number) in the parental subgenomes versus the tetraploid genome, untransformed. 2:1 and 1:1 lines shown.

N. rustica against *N. paniculata* (maternal; blue) and *N. undulata* (paternal; red).

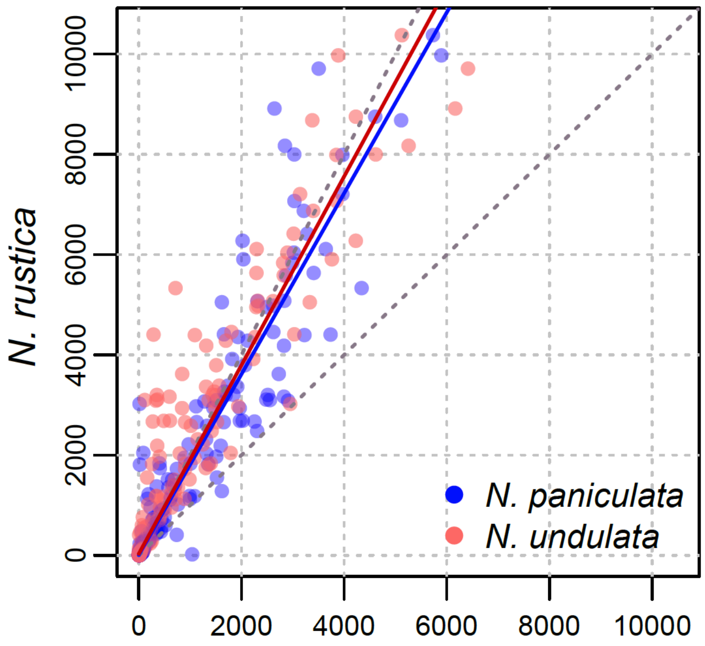

N. repanda against *N. obtusifolia* (paternal; blue) and *N. sylvestris* (maternal; red).

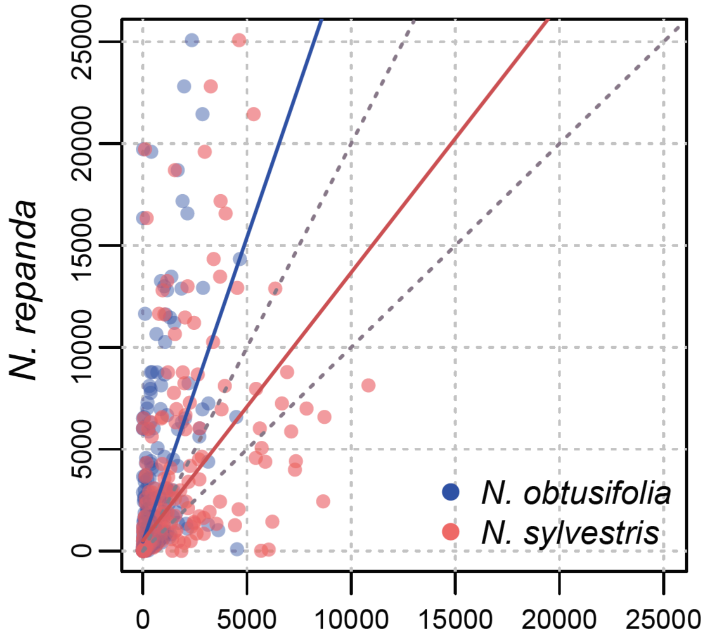

N. benthamiana against *N. noctiflora* (maternal; blue) and *N. sylvestris* (paternal; red).

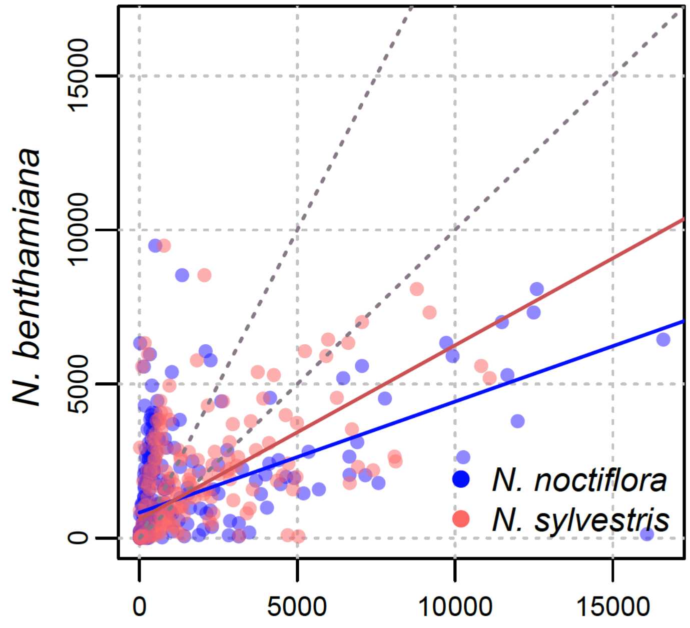

Figure S2. Regression plots with LOESS lines with a span = 0.75.

N. rustica against *N. paniculata* (maternal; blue) and *N. undulata* (paternal; red).

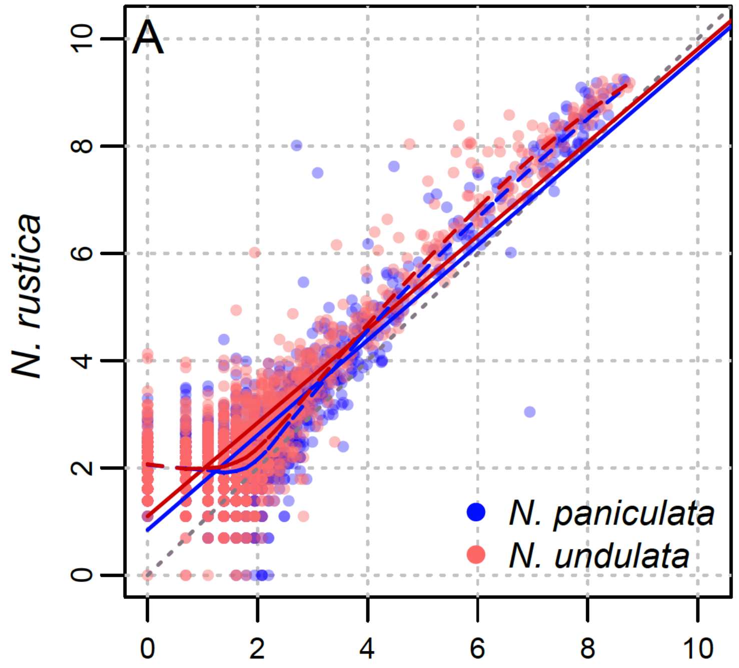

N. repanda against *N. obtusifolia* (paternal; blue) and *N. sylvestris* (maternal; red).

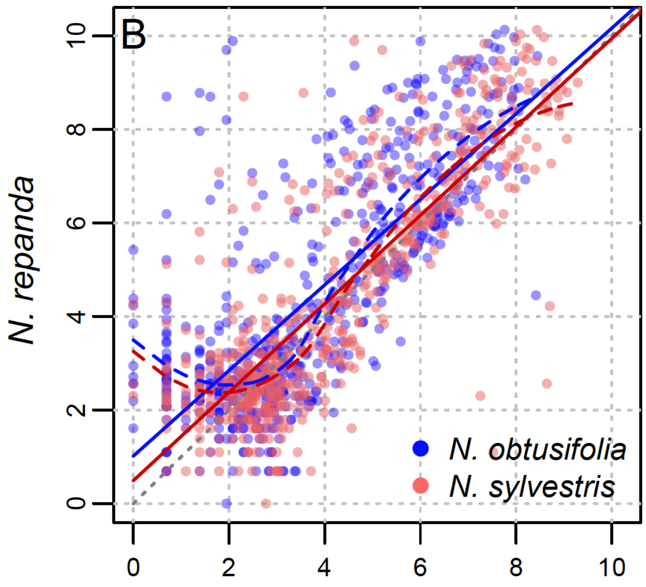

N. benthamiana against *N. noctiflora* (maternal; blue) and *N. sylvestris* (paternal; red).

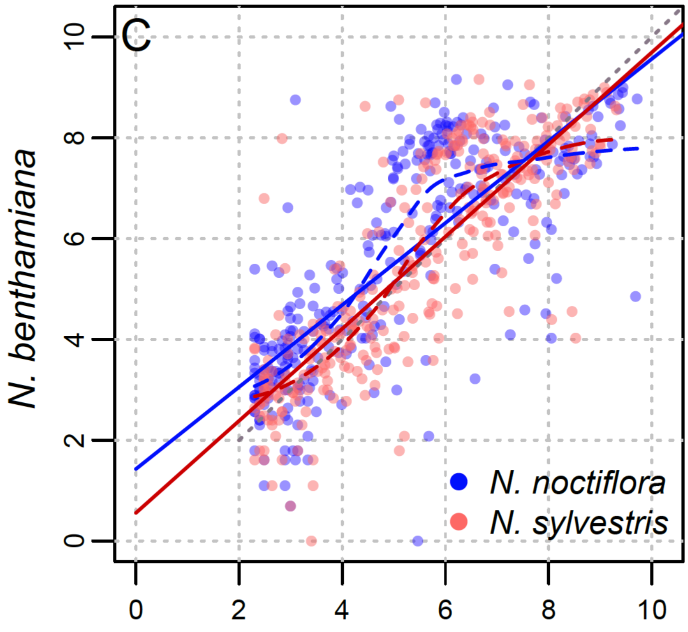

Supplement: Supplementary file 1 [file genes-11-00216-s001.pdf]
